# Gut Microbiome Structure and Association with Host Factors in a Korean Population

**DOI:** 10.1128/mSystems.00179-21

**Published:** 2021-08-03

**Authors:** Mi Young Lim, Seungpyo Hong, So-Jung Bang, Won-Hyong Chung, Ji-Hee Shin, Jung-Ha Kim, Young-Do Nam

**Affiliations:** a Research Group of Healthcare, Research Division of Food Functionality, Korea Food Research Institute, Jeollabuk-do, Republic of Korea; b Graduate School of Biotechnology and Institute of Life Science and Resources, Kyung Hee University, Gyeonggi-do, Republic of Korea; c Department of Family Medicine, Chung-Ang University Hospital, Chung-Ang University College of Medicine, Seoul, Republic of Korea; Duke University

**Keywords:** dietary pattern, enterotype, gut microbiome, host factor, host-microbiome, microbial cluster

## Abstract

Characterizing the gut microbiome in the healthy population is the first step in elucidating its associations with host health conditions. Populations with different diet patterns, lifestyles, and genetic backgrounds harbor different gut microbes. In this study, we characterized the gut microbiome of 890 healthy Koreans using 16S rRNA sequencing. The Korean population harbored a relatively large fraction of the *Prevotella* enterotype and presented a distinctive gut microbiome, compared to that in the populations of other countries. Additionally, we determined the clusters of cooccurring microbes that were quantitatively correlated with each other. We found that microbe composition of the gut was strongly associated with age. We identified that the abundance of members of *Bacteroidia* and *Clostridia* differed with the host dietary patterns, body mass index, and stool frequency. The gut microbiome data obtained in this study would be an important resource for future studies addressing microbial contributions in health and disease.

**IMPORTANCE** Comparing the gut microbiomes of healthy controls and disease patients showed that the composition of the gut microbiome is associated with various host health conditions. The gut microbiome in healthy Western populations is well characterized, while that of non-Western populations, with different diet patterns, lifestyles, and genetic backgrounds, is not clearly defined. In this study, we characterized the microbiome of 890 healthy Korean individuals using 16S rRNA sequencing and found that Koreans have a gut microbiome different from that in the individuals of neighboring countries. The members of *Bacteroidetes* and *Firmicutes* cooccurred and were quantitatively associated with each other. Additionally, we found that the gut microbial composition is strongly associated with the host’s age. The microbiome data presented here represent the gut microbiome of a healthy Korean population and could be used to unveil gut microbiome-associated host conditions in this population.

## INTRODUCTION

The gut microbiota plays a crucial role in human health and diseases, such as infections, allergies, obesity, diabetes, and even cancer (1). This has sparked interest in the study of the microbiome and its potential application for enhancing the diagnosis, prognosis, and treatment of microbiome-related diseases. However, recruiting participants, collecting fecal samples, selecting case and control groups, and analyzing the associations between microbes and diseases have been challenging. The last two problems ensue because individuals harbor a wide variety of microbes and the normal status of the microbiome is not clearly defined. Large-scale studies have been conducted to characterize the normal microbiome in various countries, such as the United States (2, 3), China (4), Belgium (5), and the Netherlands (6). The gut microbiome differs among countries (7, 8), and there are significant regional variations within a country (4). Therefore, to accurately explore microbe-host associations, it is essential to obtain microbiome data from many individuals who share similar genetic backgrounds and lifestyles. Previous studies on Koreans demonstrated that the gut microbial composition of Koreans was different from that of the Americans, Chinese, and Japanese (9, 10). However, these studies targeted a small number of Korean individuals, 19 and 20 samples. It is necessary to determine the microbial composition from a larger number of Korean samples. South Korea is a place where nearly half the population live in the metropolitan area of Seoul and have a rice-based diet enriched with fermented foods and vegetables (11). Due to these distinctive traits, it would be valuable to characterize the gut microbiome of the Korean population.

Here, we aimed to characterize the microbiome composition of the normal gut of the Korean population living in the metropolitan area of Seoul using 16S rRNA sequencing. The microbiome from healthy Korean donors was compared to that of those from other countries including neighboring countries, such as China and Japan, and the interactions among the microbes were investigated. In addition, we evaluated the influence of host factors in the development of the gut microbiome. The gut microbiome data set provided in this study delineates the gut microbial compositions of healthy Koreans, and it could be utilized as the control group for case-control studies, for understanding the interaction between the gut microbes and the host.

## RESULTS

### Taxonomic structure of the Korean gut microbiome.

We recruited 890 healthy volunteers from the Seoul metropolitan area (77.5% female; mean age [SD] of the population, 55.6 [15.0] years; see Table S1 in the supplemental material). Their gut microbiome was profiled using 16S rRNA sequencing and amplicon sequence variant (ASV) analysis. We named the ASVs after their lowest-level assigned taxonomy name and their rank of total read count. For example, Faecalibacterium prausnitzii ASV 1 is the most abundant ASV among ASVs annotated as *F. prausnitzii*, and *Bacteroides* ASV 2 is the second most abundant ASV among the ASVs classified as *Bacteroides* but not classified at the species level. Additionally, in this study, the prevalence of taxonomic groups, or ASVs, refers to the proportion of individuals having nonzero reads for a taxonomic group.

10.1128/mSystems.00179-21.4 TABLE S1 Clinical characteristics of the study participants. Download Table S1, XLSX file, 0.01 MB. Copyright © 2021 Lim et al. 2021 Lim et al. https://creativecommons.org/licenses/by/4.0/ This content is distributed under the terms of the Creative Commons Attribution 4.0 International license.

We first investigated the abundance and prevalence of the taxonomic groups. *Bacteroidetes* and *Firmicutes* were the most abundant phyla in the Korean population, accounting for 48.8% and 42.8% of the total read counts, respectively, and they were observed in all the samples (Fig. S1A). *Proteobacteria* and *Actinobacteria* constituted only 5.9% and 1.5% of the total read counts, respectively, yet these microbes were found in 99.9% and 98.3% of the study subjects, respectively (Fig. S1A). At the genus level, *Bacteroides* and *Prevotella*, which belong to the *Bacteroidetes* phylum, were the most abundant, accounting for 25.4% and 16.0% of the total read counts, respectively. *Prevotella* was observed in 81.7% of the population, while *Bacteroides* was found in all individuals. *Prevotella* was abundant in a group of individuals who have a relatively low abundance of *Bacteroides* (Spearman’s correlation coefficient = −0.74, *P* = 3.72 × 10^−152^) (Fig. S1B and C). *Faecalibacterium*, *Oscillospira*, and *Ruminococcus*, all belonging to the phylum *Firmicutes*, accounted for 13.2%, 2.6%, and 2.4% of the total reads; they were found in 99.6%, 99.6%, and 99.0% of the study individuals, respectively.

10.1128/mSystems.00179-21.1 FIG S1 Taxonomic structure of the Korean microbiome. (A) Average relative abundance and prevalence of each taxonomic unit. Each taxonomic unit is represented as a bar; the height indicates the average relative abundance, and the color indicates the prevalence. (B) Relative abundance of major taxonomic units for each individual. The relative abundances of the top-10 most abundant taxa in the Korean population were plotted as a stacked bar for each individual. The color codes for the taxa are shown in the legend. Download FIG S1, JPG file, 2.3 MB. Copyright © 2021 Lim et al. 2021 Lim et al. https://creativecommons.org/licenses/by/4.0/ This content is distributed under the terms of the Creative Commons Attribution 4.0 International license.

**FIG 1:**
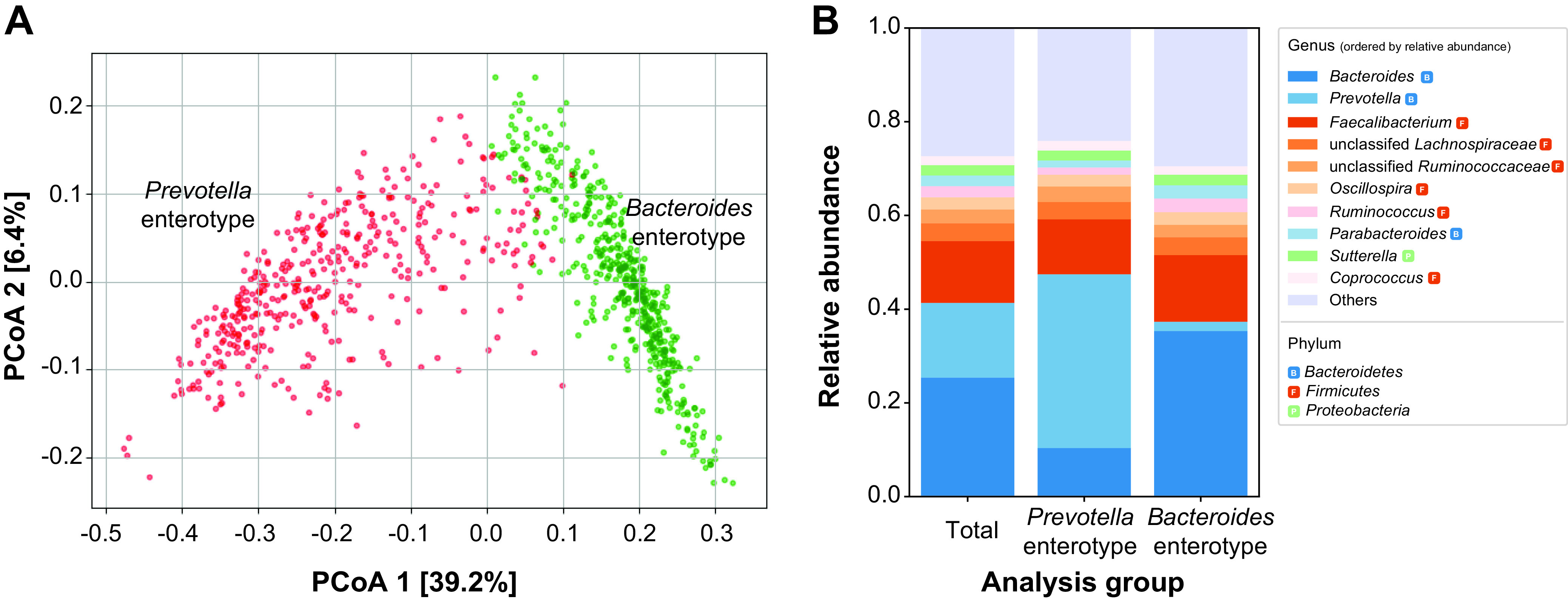
Taxonomic structure of the Korean gut microbiome. (A) Principal-coordinate analysis (PCoA) plot of the gut microbiome, based on the weighted UniFrac distance matrix. Points are colored based on the enterotype of each individual. (B) The relative abundances of the 10 most abundant genera in the Korean gut microbiome for the total samples and the two enterotypes.

We assessed the enterotype (ET) distribution of the gut microbiome of healthy donors, by clustering individuals based on their gut microbiome composition (weighted UniFrac distance). Korean individuals formed two distinct clusters, here termed *Bacteroides* ET and *Prevotella* ET, based on the dominating genus in each ET (Fig. 1A). Approximately 60% (*n* = 536) of the study subjects belonged to the *Bacteroides* ET, while the remaining 40% (*n* = 354) belonged to the *Prevotella* ET. The *Bacteroides* ET primarily consisted of *Bacteroides* (35.3%), *Faecalibacterium* (14.2%), and *Prevotella* (2%), while the *Prevotella* ET comprised *Prevotella* (37.1%), *Faecalibacterium* (11.7%), and *Bacteroides* (10.4%) (Fig. 1B).

Prevotella copri can be grouped into four clades—A, B, C, and D—depending on their genetic structure; clade A was ubiquitously found among both the Westernized and non-Westernized populations, while clades B, C, and D were predominantly found in non-Westernized populations, whose diet contains more fibers and complex carbohydrates (12). As Koreans consume more vegetables than the Western populations (11), we evaluated whether the Korean population has *P. copri* clades typical of non-Westernized populations. Consequently, we found that *P. copri* ASVs of the Korean gut microbiome mostly belonged to clade A. This indicates that the gut microbiome of the Koreans is similar to that of the Westernized populations, pertaining to the *P. copri* clade (Table S2).

10.1128/mSystems.00179-21.5 TABLE S2 List of Prevotella copri ASVs and their clade. The sequences of *P. copri* ASVs were queried against the genome of the clades annotated by Tett et al. (A. Tett, K. D. Huang, F. Asnicar, H. Fehlner-Peach, et al., Cell Host Microbe 26:666–679.e7, 2019, https://doi.org/10.1016/j.chom.2019.08.018). The genomes that perfectly matched to the *P. copri* ASVs are listed along with their clades. Download Table S2, XLSX file, 0.01 MB. Copyright © 2021 Lim et al. 2021 Lim et al. https://creativecommons.org/licenses/by/4.0/ This content is distributed under the terms of the Creative Commons Attribution 4.0 International license.

### Korean microbiome in the context of that of other countries.

We compared the gut microbiome from the Korean population to the gut microbiomes of the populations from other countries. Data sets from China (two data sets with 412 and 25 samples) (13, 14), Japan (*n* = 468) (15), Spain (*n* = 40) (16), Chile (*n* = 41) (17), and Nigeria (*n* = 48) (18) were analyzed. Only data sets targeting the V3-V4 region of the 16S rRNA gene were included in the analysis to minimize methodological artifacts.

The overall structure of the Korean microbiome was compared to that of the samples from each of the other countries using permutational multivariate analysis of variance (PERMANOVA) tests. The overall structure of the Korean gut microbiome varied from that of each non-Korean gut microbiome (PERMANOVA, *P* < 0.001, for each of the six data sets). The differences in the overall structure may originate from the differences in their substructures, such as enterotypes. Therefore, we compared the enterotype ratio of the Korean gut microbiome to that of the populations from the other countries. We evaluated for the similarity between the samples, both Korean and others, belonging to the same enterotype.

**FIG 2:**
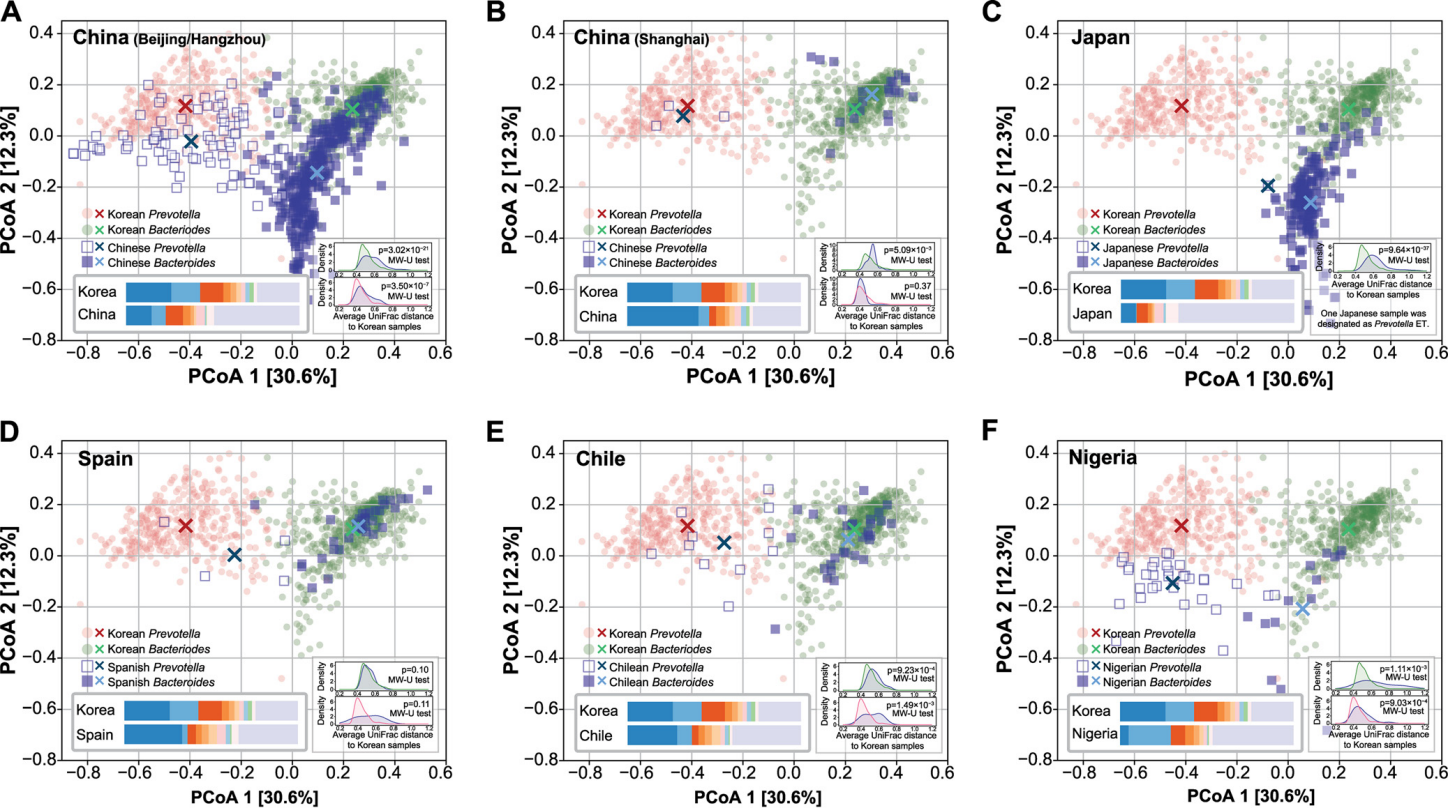
Comparison of the gut microbiome from the Korean population with the gut microbiomes of the populations from other countries. Principal-coordinate analysis (PCoA) plots of gut microbiomes from Korean and (A) Chinese (Beijing/Hangzhou), (B) Chinese (Shanghai), (C) Japanese, (D) Spanish, (E), Chilean and (F) Nigerian populations. Green and red dots represent Korean samples with the *Bacteroides* and the *Prevotella* ET, respectively. The samples of other countries are represented by filled blue squares and empty blue squares for *Bacteroides* and *Prevotella* ET, respectively. The centers of the Korean samples and the other country’s samples were marked with X’s. The left bottom inset illustrates the relative abundances of the 10 most abundant genera in the Korean gut microbiome, using the same color code described in Fig. 1A. The distribution of average weighted UniFrac distances between samples is plotted in the right bottom inset. The distribution between Korean *Bacteroides* (green) and *Prevotella* (red) ET samples was plotted in the upper and lower panels, respectively. The distributions between the Korean samples and the samples from the other countries are shown in blue.

In the principal-coordinate analysis (PCoA) plots, a larger number of samples from other countries were closer to the Korean *Bacteroides* ET samples than to the Korean *Prevotella* ET (Fig. 2). Therefore, we evaluated whether the ET distribution of the Korean population is statistically different from that of the populations from the other countries. Each sample of non-Korean origin was classified as *Bacteroides* ET or *Prevotella* ET, based on the ET of the Korean sample with the shortest UniFrac distance to the analyzed sample. We found that the proportion of Korean *Bacteroides* ET samples (60%) was lower than that of samples with Chinese origin, from both Beijing/Hangzhou (79.4%, chi-squared test *P* = 1.66 × 10^−11^) and Shanghai (88.0%, 9.32 × 10^−3^), and the samples from Japanese (99.4%, 1.20 × 10^−23^), Spanish (90.0%, 2.95 × 10^−4^), and Chilean (73.2%, 0.13) populations. However, the proportion of *Bacteroides* ET samples in the Nigerian samples was significantly lower (30%, 6.93 × 10^−5^) than that of the Korean samples.

There are two enterotypes; and therefore, the microbiome can be thought of as a mixture of two different microbial structures. Therefore, we separated the samples based on their enterotype and compared the microbial structure within the same enterotype. First, we compared the Korean *Bacteroides* ET samples with the corresponding samples from the geographic neighbor countries, China and Japan. The overall microbial structure of Korean *Bacteroides* ET samples was different from that of *Bacteroides* ET samples from each of the Chinese and Japanese samples (PERMANOVA, *P* < 0.001, for each of the three data sets). However, the center of the Korean samples was distant from that of the Beijing, Hangzhou, and Japanese samples, but it was close to the center of the samples from Shanghai (Fig. 2A, B, and C). Further, we evaluated whether the samples from other countries are different from those from the Korean population within each enterotype, by comparing the distributions of the average distance between these countries’ samples and the Korean samples and those between the Korean samples. A significant number of *Bacteroides* ET samples from Beijing/Hangzhou residents had a longer distance from the Korean *Bacteroides* ET samples, compared to the distances between the Korean *Bacteroides* ET samples (Mann-Whitney *U* test, *P* = 3.02 × 10^−21^) (Fig. 2A). However, the distances between a part of *Bacteroides* ET samples from Beijing/Hangzhou residents and the Korean *Bacteroides* ET samples were relatively small. The distance distribution of Shanghai residents was more similar to that of the Korean samples than that of the Beijing/Hangzhou residents; however, it was statistically significantly different from that of the Korean samples (Mann-Whitney *U* test, *P* = 5.09 × 10^−3^) (Fig. 2B). Japanese samples rarely overlapped Korean samples on the PCoA plot, and a significant number of Japanese *Bacteroides* ET samples were dissimilar from the Korean *Bacteroides* ET samples (Mann-Whitney *U* test, *P* = 9.64 × 10^−37^) (Fig. 2C). The microbial composition of the Japanese population was considerably different from that of the Korean population. The Japanese gut microbiome possessed only a small fraction of genera that were abundant in the Korean gut microbiome, while having high proportions of *Blautia* (17.0%) and *Bifidobacterium* (11.1%), which were rarely observed in the Korean gut microbiome (0.8% and 1.4%, respectively) (Fig. 2C).

We compared the Korean *Bacteroides* ET samples to those from Spanish, Chilean, and Nigerian populations. The overall microbial structure of the Korean *Bacteroides* ET samples was significantly different from that of the *Bacteroides* ET samples from each of the other countries (PERMANOVA, *P* < 0.001, for each of the three countries). However, the centers of samples from Spaniards and Chileans were located close to the center of samples from the Koreans, while the center of Nigerian samples was distant from that of the Korean samples (Fig. 2D, E, and F). We statistically compared the distances to the Korean samples, and the distance distribution was similar to that among the Korean population (Mann-Whitney *U* test, *P* = 0.10) (Fig. 2D). This suggests that the microbial compositions of the Spanish and Korean individuals belonging to *Bacteroides* ET would be similar, despite their large geographical distance and ethnic differences. Statistically, a slightly larger number of Chilean *Bacteroides* ET samples were more dissimilar to the Korean *Bacteroides* ET samples (Mann-Whitney *U* test, *P* = 9.23 × 10^−4^) (Fig. 2E). A part of the Nigerian *Bacteroides* ET samples possessed microbial compositions similar to that of the Korean *Bacteroides* ET samples, but when the entire set of samples was considered, they were significantly different from that of the Korean *Bacteroides* ET samples (Mann-Whitney *U* test, *P* = 1.11 × 10^−3^) (Fig. 2F).

We compared the Korean *Prevotella* ET samples with that from each of the other countries. The Korean *Prevotella* ET samples were significantly different from the Chinese (Beijing/Hangzhou), Chilean, and Nigerian *Prevotella* ET samples (PERMANOVA, *P* < 0.001, for each of the three data sets). There were only a small number of *Prevotella* ET samples from Chinese (Shanghai), Japanese, and Spanish populations, and therefore, it is unclear whether the *Prevotella* ET samples of these populations are similar or dissimilar to those of the Korean population.

### Microbial clusters of Korean gut microbiome.

**FIG 3:**
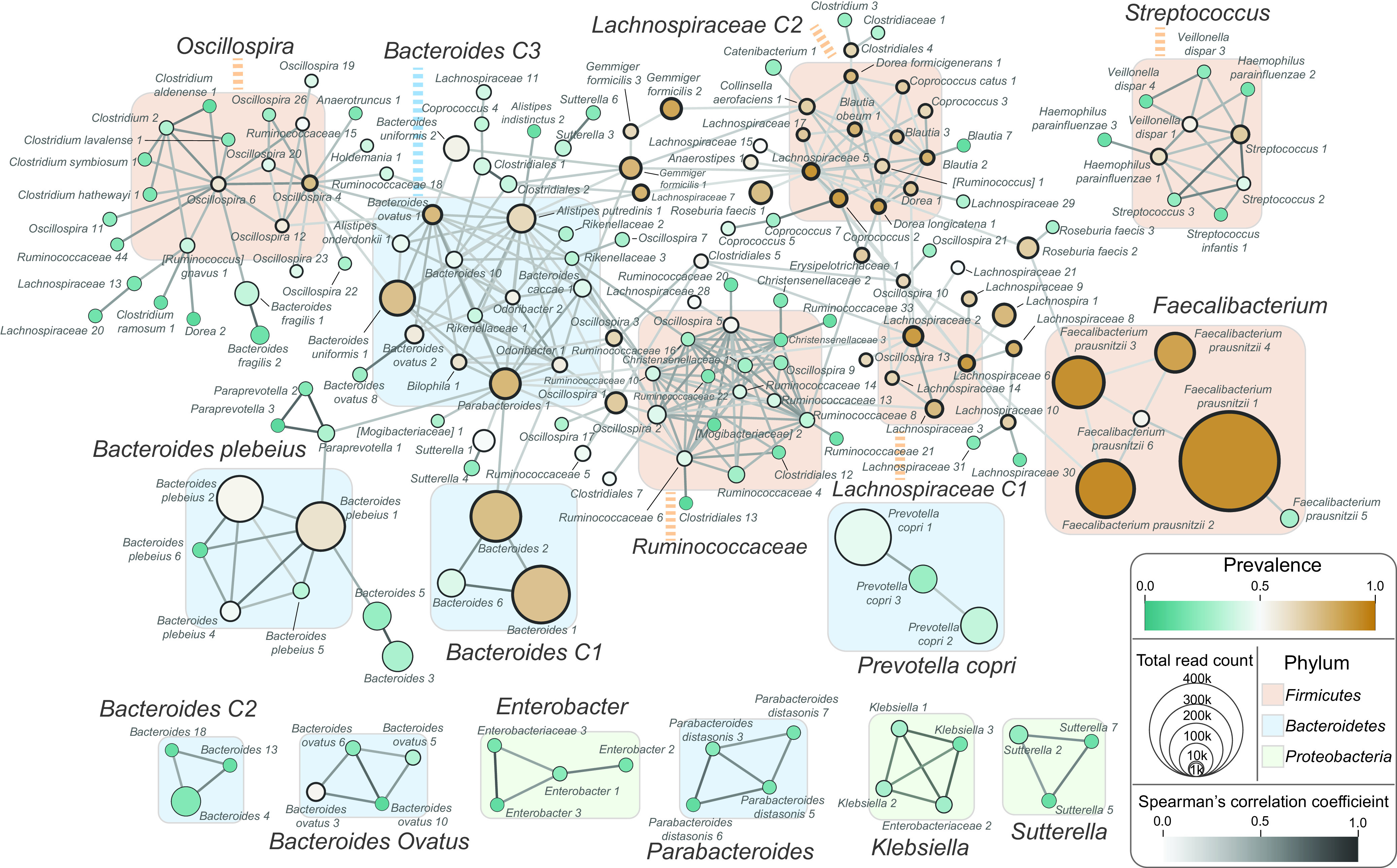
ASV clusters of the Korean gut microbiome. The association structure of ASVs is represented as a network of ASVs using ASVs as nodes and associations between ASVs as edges. The association between two ASVs is evaluated based on their cooccurrence (Fisher’s exact test, FDR < 10^−6^) and positive quantitative association (Spearman’s correlation test, FDR < 10^−3^). The graphs with more than three nodes are shown. The nodes are colored in green to orange according to the prevalence of the ASVs. The total number of reads is depicted by the size of the node. The minimal size of the node is fixed for the clear display of nodes. The nodes, or ASVs, are labeled only with the taxonomy name and its read count rank. The colors of the edges represent the Spearman correlation coefficient of the association. ASVs are grouped into microbial clusters based on the connectivity in the network and their phylum, and the clusters are named after their abundant members. The members of each cluster mainly belong to one of the three phyla, and the background box of the cluster is colored accordingly.

We investigated the interactions among the microbes that constitute the Korean gut microbiome. Such interactions can be determined by the statistical analysis of microbial abundance correlations and niche overlapping (19–21). We determined the pairs of ASVs, which were present in the same sample with positively correlated abundances. These ASV pairs were used to construct the interaction network of ASVs. In this network, ASVs within the same phylum tended to be connected to each other (Fig. S2). We grouped those ASVs and defined them as microbial clusters, which were named after the taxonomic name of the most abundant ASV in the cluster (Fig. 3).

10.1128/mSystems.00179-21.2 FIG S2 Phylum of ASVs in the microbial clusters. The ASVs in the interaction network are colored based on their phylum. The size of the node represents the total read counts of each ASV. Download FIG S2, JPG file, 2.3 MB. Copyright © 2021 Lim et al. 2021 Lim et al. https://creativecommons.org/licenses/by/4.0/ This content is distributed under the terms of the Creative Commons Attribution 4.0 International license.

The ASVs that belonged to *Firmicutes* interacted with each other and formed six microbial clusters (Fig. 3 and Fig. S2). The *Faecalibacterium* cluster consisted of *F. prausnitzii* ASVs that were highly abundant and prevalent in the Korean microbiome, but the ASVs in this cluster were weakly correlated with each other (Fig. 3). *Lachnospiraceae*, *Ruminococcaceae*, *Oscillospira*, and Streptococcus clusters were composed of ASVs belonging to *Firmicutes* (Fig. 3 and Fig. S2). The ASVs of these clusters were less in abundance but were highly connected to each other.

ASVs from *Bacteroidetes* formed seven microbial clusters. The *Bacteroides* C1 and C2 clusters were composed of ASVs annotated as the same genus, *Bacteroides*; however, the two clusters were disjointed in the network (Fig. 3). *Bacteroides* ASVs in the *Bacteroides* C1 cluster were highly prevalent and highly abundant, while those in the *Bacteroides* C2 cluster were less prevalent and less abundant. The *Bacteroides* C3 cluster was composed of three *Bacteroides* ASVs, B. uniformis, B. ovatus, and B. caccae, and others including *Parabacteroides* and Alistipes putredinis. Prevotella copri, Bacteroides ovatus, and *Parabacteroides* clusters comprised ASVs annotated to the same species (Fig. 3). The *Proteobacteria* ASVs formed three separated clusters in the network, Enterobacter, Klebsiella, and *Sutterella* (Fig. 3).

We further investigated the pairs of microbes whose abundances were positively correlated with each other, regardless of their cooccurrence. The abundances of ASVs belonging to the same phylum were strongly correlated (Fig. S3A and B). However, a relatively small number of ASVs were negatively correlated, and such associations were found mostly between *Bacteroides* ASVs and *P. copri* ASVs (Fig. S3C).

10.1128/mSystems.00179-21.3 FIG S3 Quantitative microbial association. (A and B) ASV pairs with Spearman’s correlation coefficients larger than 0.3 are represented as interaction networks. The nodes represent ASVs, and the edges represent their statistical association. The color of a node stands for the prevalence (A) and phylum (B) of the ASV. The edge colors represent the Spearman correlation coefficients. (C) Quantitatively and negatively associated ASVs (FDR < 10^−3^). The color of a node stands for the phylum of the ASV. Download FIG S3, JPG file, 2.6 MB. Copyright © 2021 Lim et al. 2021 Lim et al. https://creativecommons.org/licenses/by/4.0/ This content is distributed under the terms of the Creative Commons Attribution 4.0 International license.

### Demographic differences in the Korean gut microbiome.

**FIG 4:**
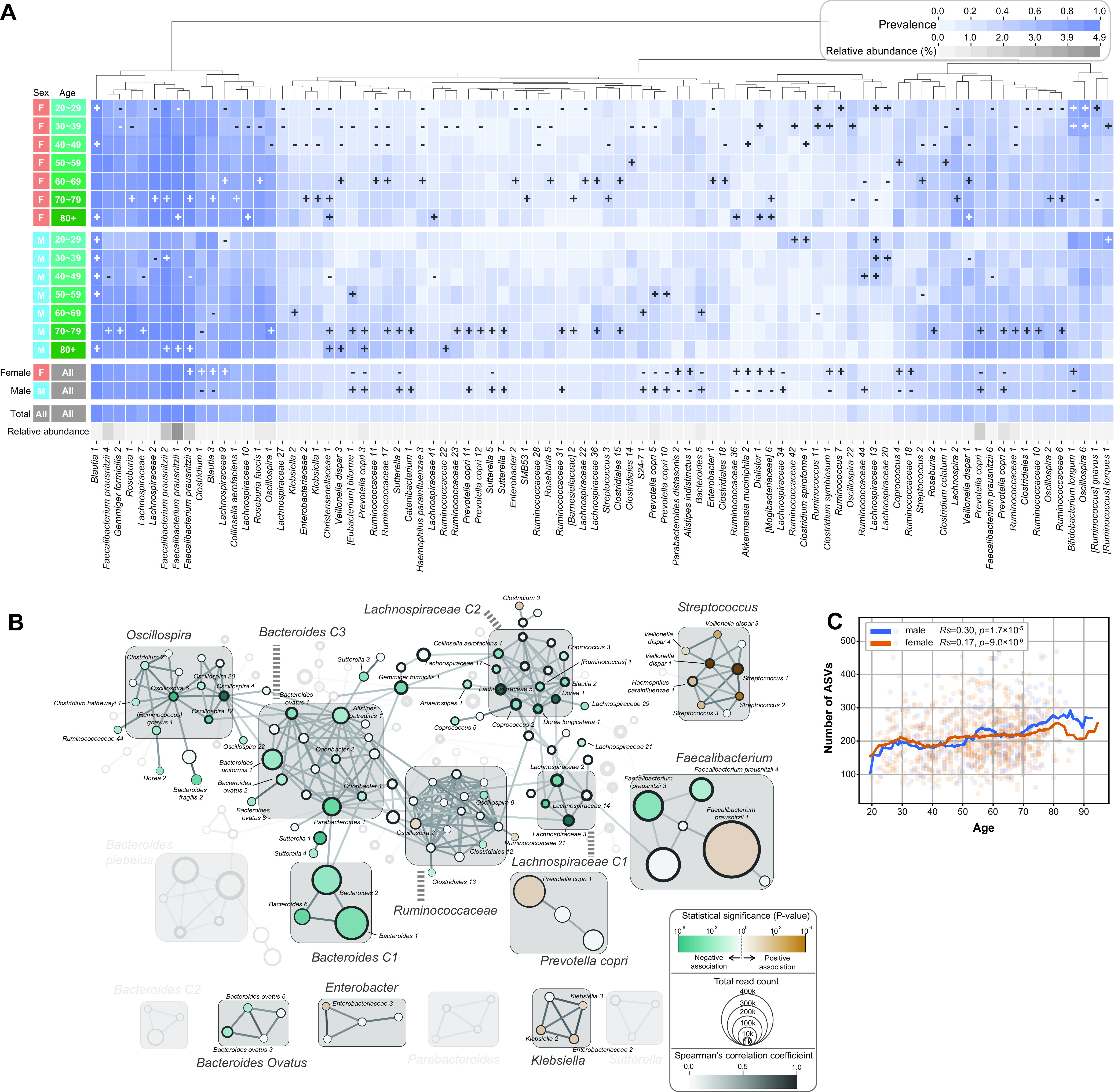
Microbes in different demographic groups. (A) The prevalence of each ASV in a specific demographic group is evaluated using hypergeometric distribution, and those showing statistically significant difference (FDR < 0.1) are marked with “+” or “−”, when their prevalence is larger or smaller than that of the entire group, respectively. The microbes that are different in fewer than two demographic groups are not shown. The last row shows the relative ASV abundance. (B) Spearman’s correlations between ASV abundance and age are calculated, and those with FDRs of <0.2 are plotted. (C) The age of the host is correlated with the microbial diversity (number of ASVs) in both male and female populations. The moving averages of diversities in window sizes of 10 years are plotted as lines for the male and the female populations, and the Spearman correlation coefficient and corresponding *P* values are listed in the upper left box.

We investigated the host-gut microbe interactions by analyzing the statistical association between host factors and the prevalence and abundance of ASVs. At first, the associations of the gut microbiome with the two major demographic variables, age and sex, were evaluated. A number of ASVs, such as *F. prausnitzii*, were more prevalent in the older groups than in the younger groups, regardless of sex (hypergeometric distribution, false-discovery rate [FDR] < 0.1) (Fig. 4A). Age positively correlated with the alpha diversity in both the sexes (Spearman’s correlation test, male *P = *1.7 × 10^−5^, female *P = *9.0 × 10^−6^) (Fig. 4C). However, some ASVs like Bifidobacterium longum 1 ASV and *Oscillospira* 6 ASV were more prevalent in the youngest populations (Fig. 4A). Correlation analysis between age and ASV abundance revealed that the abundances of ASVs in *Lachnospiraceae* C1 and C2, *Oscillospira*, *Bacteroides* C1, and *Bacteroides* C3 clusters decreased with age while ASVs’ abundance in Streptococcus, *P. copri*, and Klebsiella clusters increased with age (Fig. 4B). The difference in the prevalence of ASVs between the male and female individuals was smaller than that observed among the different ages; however, *P. copri* ASVs and *Sutterella* ASVs were more frequently found in males, while *Clostridium* ASV 1 and Clostridium symbiosum ASV 1 were more frequently observed in females (hypergeometric distribution, FDR < 0.1) (Fig. 4A).

### Association of gut microbiome with clinical and lifestyle features.

We examined the associations of ASV abundance with host health status and lifestyle factors. During the analysis of the microbe-host factor association, the contribution of age was considered if the age was significantly associated with the host factor (Wald test). A number of ASVs were significantly associated with host factors including stool frequency, waist circumference, body mass index (BMI), and food intake patterns (FDR < 0.2) (Table S3).

10.1128/mSystems.00179-21.6 TABLE S3 Number of ASVs significantly associated with host factors. Download Table S3, XLSX file, 0.01 MB. Copyright © 2021 Lim et al. 2021 Lim et al. https://creativecommons.org/licenses/by/4.0/ This content is distributed under the terms of the Creative Commons Attribution 4.0 International license.

**FIG 5:**
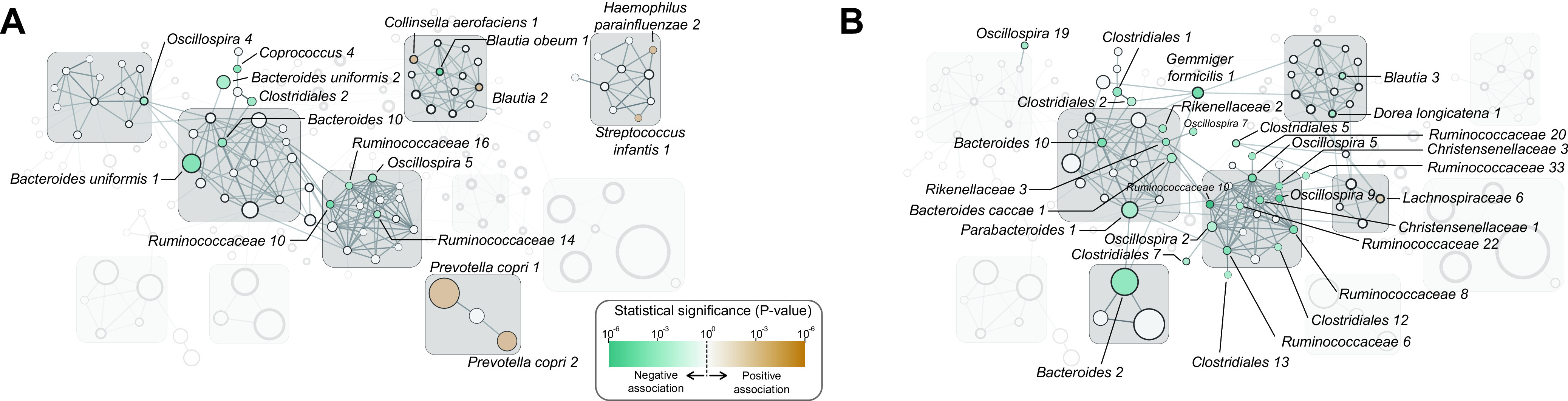
Microbial association with host features. The ASVs significantly associated with BMI (A) and stool frequency (B) are colored according to their statistical significance.

The ASVs that showed significant associations with BMI and stool frequency were analyzed. The abundances of *P. copri* 1 and *P. copri* 2 ASVs, in the *P. copri* cluster, were positively associated with BMI, while Bacteroides uniformis 1 and *Bacteroides* 10 ASVs, in the *Bacteroides* C3 cluster, and the four ASVs in the *Ruminococcaceae* cluster were negatively associated with BMI (Fig. 5A). In the *Lachnospiraceae* C2 cluster, the abundance of Blautia obeum 1 ASV was negatively associated with BMI, but that of *Blautia* 2 and Collinsella aerofaciens 1 ASVs were positively correlated with BMI (Fig. 5A). The abundances of 11 ASVs of the *Ruminococcaceae* cluster and five ASVs of the *Bacteroides* C3 cluster were negatively associated with stool frequency (Fig. 5B).

### Association of gut microbiome with dietary pattern.

**FIG 6:**
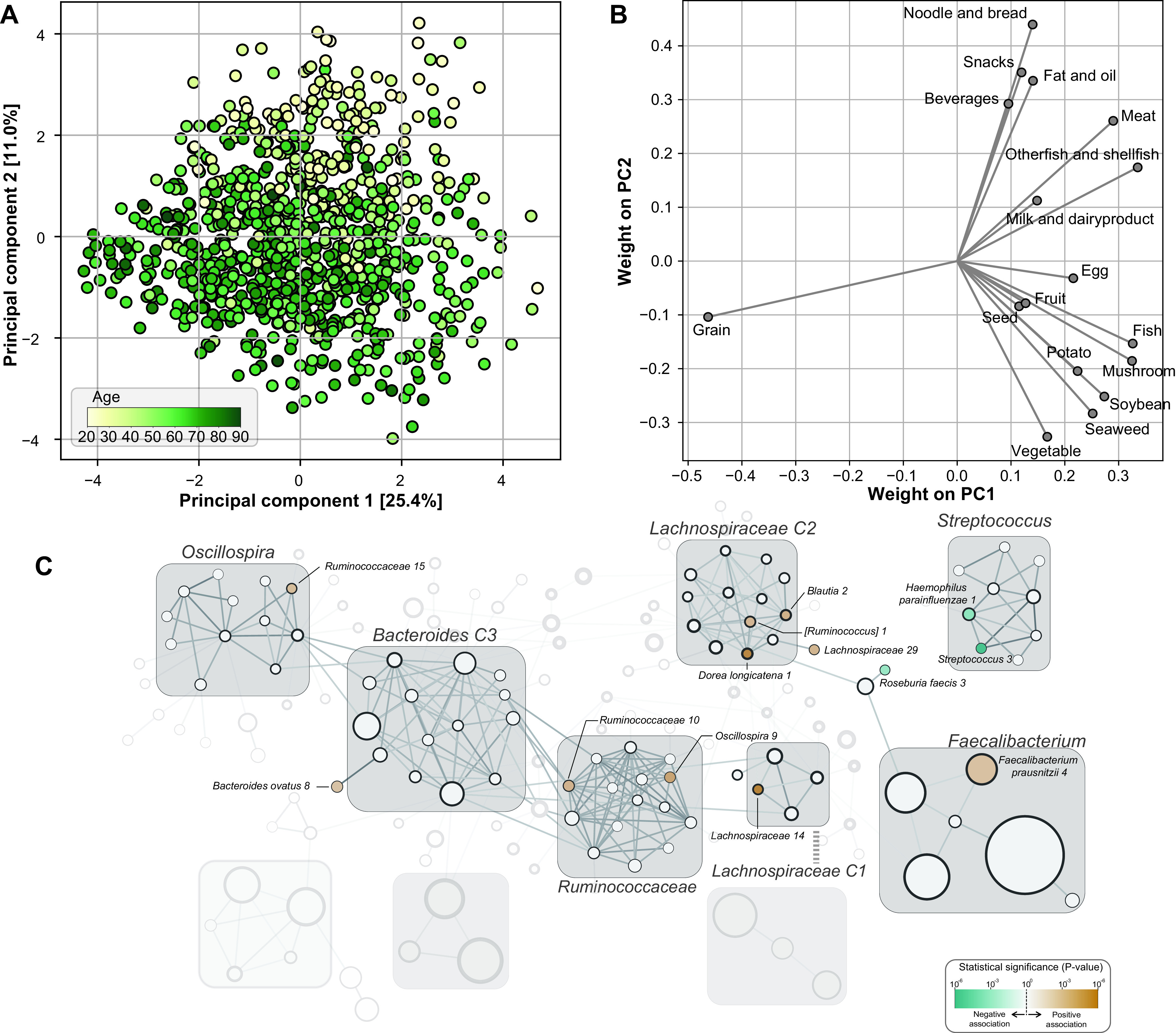
Diet pattern and associated microbes. (A) The principal-component analysis (PCA) for diets of the participants. Dots are colored by age. (B) The weights of each food group in the first and the second principal components. (C) The ASVs significantly associated with the second principal component of the dietary pattern are colored with their statistical significance.

The microbial composition is affected by the diet of the host (22, 23); we evaluated which microbes were affected by this factor. A relatively small number of ASVs was associated with a single dietary group (Table S3). However, 17 and 3 ASVs were associated with the second and the first principal components of dietary pattern, respectively (Table S3). The first principal component (PC1) was largely dominated by grain intake, while the second principal component (PC2) differentiated the Westernized diet—marked by the intake of noodles, snacks, and meat—from the traditional diet—marked by vegetables, seaweed, and soybean intake—and was negatively associated with age (Spearman’s correlation coefficient = −0.51, *P* = 3.54 × 10^−61^) (Fig. 6A and B). Three ASVs were positively associated with the diet PC1 at an FDR of <0.2: *Sutterella* ASV 4 (Wald test, *P* = 3.4 × 10^−4^), *Coprococcus* ASV 6 (*P* = 5.3 × 10^−4^), and *Paraprevotella* ASV1 (*P* = 5.8 × 10^−4^). The diet PC2 was positively associated with *Lachnospiraceae* ASV 14 (*P* = 6.3 × 10^−6^) and Dorea longicatena ASV1 (*P* = 9.6 × 10^−6^) and negatively associated with Streptococcus ASV 3 (*P* = 4.0 × 10^−6^) and Haemophilus parainfluenzae ASV 5 (*P* = 8.7 × 10^−4^) (Fig. 6C).

## DISCUSSION

We characterized the gut microbial compositions of healthy donors of the Korean population living in the Seoul metropolitan area using 16S rRNA sequencing. The Korean gut microbiome consisted of two enterotypes, *Bacteroides* and *Prevotella*. The Korean diet includes a wide variety of vegetables; therefore, we investigated whether the high abundance of *P. copri* ASVs in the Korean population was linked to the *Prevotella* clades B, C, and D, which are predominantly found in the hunter-gatherer societies that consume abundant fiber and complex carbohydrates (12). The *P. copri* ASVs in the Korean gut microbiome predominantly belonged to clade A, indicating that the Korean gut microbiome was similar to the gut microbiome of Westernized populations. Although the Korean diet consists of various side dishes, including a variety of vegetables, the staple is boiled rice, and therefore, the consumption of fiber and complex carbohydrates might not be sufficient to foster the growth of *Prevotella* clades B, C, and D.

The overall structure of the Korean microbiome was different from that of the microbiomes from the other countries; specifically, the Korean gut microbiome has a lower proportion of *Bacteroides* ET and a higher proportion of *Prevotella* ET than those in the microbiomes from other countries, with the exception of that from Nigeria. There were two distinct enterotypes in the Korean gut microbiome; therefore, we further compared the microbial structures of each enterotype individually. The overall structure of the Korean *Bacteroides* ET samples was still significantly different from that of the samples from other countries. However, the average distance distribution between the Korean samples and the samples from Spaniards was not significantly different from that among the Korean samples. Considering that the Korean, Chinese, and Japanese populations are genetically closer to each other, compared to the European population (24), there is a similarity in the microbial composition between the Koreans and Spaniards, and a dissimilarity among the Korean, Chinese, and Japanese populations. This suggests that the microbial composition could be more strongly influenced by the nongenetic host factors, such as dietary intake pattern, health status, and lifestyle.

Groups of ASVs of the Korean gut microbiome were preferentially observed in the same individuals, and they were positively correlated quantitatively. Some of the groups were composed of ASVs annotated to the same taxonomic group. However, each member of the group could be associated differently with the host factors. Considering that the microbes annotated as the same species could harbor different sets of genes (25), the differential association suggested that the microbes represented by different ASVs could have different functions and various effects on host health. Therefore, it is important to characterize the genomes of these ASVs and investigate their functionalities.

Among the host factors, age was positively associated with the diversity of the gut microbiome, and the prevalence of a number of ASVs increased with age. These results suggest that the human gut may retain newly introduced microbes and gradually expand its microbial repertoire. There are several examples showing the positive associations between microbiome diversity and age in various populations. In European adults (age 20 to 69), age was the nongenetic host factor that had the strongest positive correlation with microbiome diversity (26). In the Japanese population, the microbiome diversity was steadily maintained between ages 20 and 30 and then it increased between ages 40 and 90 (15). However, the observed age-associated diversity increment plateaued after the age of 40 among the adults from the United States, the United Kingdom, and Colombia (27). These results indicate that microbial diversity increases with age but that the patterns of increase across the age groups vary, depending on the study population. A meta-analysis of the gut microbiome from various populations would help resolve the relationship between microbial diversity and age.

The biological sex of a host can affect the host-gut microbe interactions. However, the ASV-host factor association analysis, performed separately on the female and male populations, indicated a smaller number of associations in the male group than in the female group (see Table S3 in the supplemental material). In the female samples, 28 and 14 ASVs were associated with BMI and stool frequency, respectively, but there was no statistically meaningful association with the male samples. This could be attributed to the sample size of the male group (*n* = 200), which was not sufficient to reveal statistically significant associations between ASVs and host factors; we were able to find only relatively weak associations even when using the total data. The evaluation of a higher number of male samples is necessary to accurately analyze sex-related associations.

The abundances of ASVs were associated with host factors such as BMI, stool frequency, and diet. BMI was positively associated with *P. copri* cluster ASVs but negatively associated with *Bacteroides* and *Ruminococcaceae* cluster members. The positive correlation between *P. copri* and BMI is consistent with the report that the family *Prevotellaceae* is enriched in obese individuals (28). *Ruminococcaceae* can protect the host from weight gain (29). The members of *Ruminococcaceae* are well-known butyrate producers, along with *Lachnospiraceae* and *Bacteroidetes* (30); short-chain fatty acids can reduce lipogenesis and ameliorate obesity (31). Therefore, it should be evaluated whether the reported ASVs could lower the BMI. The abundances of 11 *Ruminococcaceae* cluster ASVs were negatively associated with stool frequency. This observation is consistent with the previously reported negative association of stool frequency with unclassified *Ruminococcaceae* (32). Considering that stool frequency is negatively associated with colonic transit time (33), increased colonic transition time or low stool frequency would increase the abundance of *Ruminococcaceae*. This is consistent with the previous reports that the abundance of *Ruminococcaceae* is positively correlated with colonic transit time (34) and that harder stool (indicative of longer transit time) is associated with a higher abundance of *Ruminococcaceae* (35). A faster transit supports fast-growing bacteria (36). The abundance of *Ruminococcaceae* significantly increases with the decrease in the dilution rate in a continuous fecal microbiota culture (36), indicating that *Ruminococcaceae* is slow growing, and therefore, the increased colonic transit time supports the growth of *Ruminococcaceae*. Further studies are required to elucidate how colonic transit rate modulates the growth of specific bacteria.

Considering that nearly 900 individuals were examined in this study, only a small number of microbe-host factor associations were found. This could be because this study was designed to determine the characteristics of the gut microbiome of a healthy population. The data set was obtained from the general healthy population; therefore, the distribution of each host factor would represent that of healthy people, lacking the biased values of a specific group of individuals, or patients. We believe that our data set could be used as a control data set in future studies that assess disease-associated gut microbes. We have labeled the ASVs up to species level. However, the taxonomic classification of microbes using a single marker (V3-V4 region of 16S rRNA) could be inaccurate. Therefore, further studies using metagenomics sequencing are necessary to classify microbes at the species and strain levels (37).

In this study, we reported the characteristics of the gut microbiomes of a healthy Korean population, especially focusing on ASVs and microbial clusters. Additionally, we found their associations with age, host dietary pattern, BMI, and stool frequency. We also demonstrated that the microbiome of the Korean population is different from that in the populations from other countries, including a higher proportion (40%) of the *Prevotella* enterotype in the Korean samples. These findings emphasize the importance of microbiome data collection in each country for developing microbiome-based diagnosis or therapeutics for a specific population. We believe that the data set from this study could be used as a control in future studies aiming to explore the role of gut microbes in health and disease.

## MATERIALS AND METHODS

### Study population.

Healthy subjects were recruited from the health care center of Chung-Ang University Hospital (Seoul, South Korea). The exclusion criteria included individuals who were administered antibiotics within 3 months before the start of the study, those who had a history of major gastrointestinal surgery or active uncontrolled gastrointestinal disorders or diseases, or those diagnosed with a cancer or a chronic clinically significant cardiovascular, pulmonary, renal, or hepatic disease. Women who were pregnant or lactating were also excluded. In total, 897 volunteers were recruited, but data from seven participants were excluded from the analysis due to poor 16S rRNA sequencing results that did not pass the quality threshold described under “ASV analysis” below.

### Metadata and sample collection.

All participants underwent blood tests and anthropometrical measurements at the clinic. They also completed a questionnaire addressing their lifestyle and clinical history and a food frequency questionnaire covering 106 food items (38). The daily intake of each food item was calculated based on the intake frequency and portion size indicated in the questionnaire and then divided into 17 food groups. Among the metadata, quantitative categorical attributes were mapped to numeric values, and nominal categorical attributes were converted into Boolean variables using one-hot encoding. The entire list of 82 metadata variables used in this study is shown in Table S4 in the supplemental material. The blood test data of two participants were partially missing, and they were excluded from the associational analysis.

10.1128/mSystems.00179-21.7 TABLE S4 Metadata variables used in this study. Download Table S4, XLSX file, 0.01 MB. Copyright © 2021 Lim et al. 2021 Lim et al. https://creativecommons.org/licenses/by/4.0/ This content is distributed under the terms of the Creative Commons Attribution 4.0 International license.

The participants collected fecal samples at home within 48 h of the study using the OMNIgene-GUT tubes (DNA Genotek, Ottawa, Canada), according to the manufacturer’s instructions.

### DNA extraction and 16S rRNA sequencing.

DNA extraction and 16S rRNA sequencing were performed as described previously (39). Briefly, DNA was extracted from the fecal samples using a QIAamp DNA stool minikit (Qiagen, Hilden, Germany). Each fecal sample (250 μl) was transferred to a 2-ml tube containing 0.3 g sterile 0.1-mm zirconia beads (BioSpec, Bartlesville, OK, USA) and 1.2 ml ASL lysis buffer (Qiagen). After vortexing for 3 min, the samples were heated at 95°C for 15 min and then bead beaten twice at a frequency of 30 Hz for 1 min using a Qiagen TissueLyser II sample disruptor. After centrifuging, 1.2 ml of the supernatant was treated with an InhibitEX tablet. The subsequent DNA extraction steps were performed using a QIAcube system (Qiagen). The extracted DNA samples were stored at −20°C until use. Library construction of the V3-V4 hypervariable regions of the bacterial 16S rRNA gene was performed following the 16S metagenomic sequencing library preparation Illumina protocol (part no. 15044223 rev. B; Illumina, San Diego, CA, USA). Amplicon libraries for each sample were pooled at equimolar quantities, and they were sequenced using a MiSeq 2 × 300 instrument (Illumina).

### Bioinformatics analysis.

**(i) ASV analysis.** Sequencing data for the 16S rRNA gene were converted into a frequency table of ASVs. Briefly, we filtered out short sequences that did not match either the forward or reverse primer sequence, by aligning primer sequences onto the short reads and removing the matched region. The short reads with five or more mismatches to the primer sequence were also excluded. An ASV table was generated using the DADA2 pipeline (40) of the QIIME2 software (version 2019.01) (41), with forward and reverse truncation lengths of 270 and 220, respectively. Samples with >10,000 reads were included in the analysis. Default values were used for the other options.

**(ii) Taxonomy analysis.** Taxonomical classifications were annotated to the ASVs using the naive Bayesian classifier (42) trained on V3-V4 fragments of the Greengenes 13_8 99% operational taxonomic units (43). The prevalence of each taxonomic group, or ASV, was calculated as the number of samples with nonzero reads for a taxonomic group, divided by the total number of samples. For each sample, the relative abundance of each taxonomic group was calculated as the number of reads for the taxonomic group divided by the total number of reads of that sample.

**(iii) Diversity analysis and enterotype detection.** Alpha-diversity and principal-coordinate analysis were performed using the “diversity” (core-metrics-phylogenetic) module of the QIIME2 platform (41). Among the beta-diversity measures, the results based on weighted UniFrac distances were used in this study. The *Adonis* function in the Vegan package (version. 2.5-6) was used for the permutational multivariate analysis of variance (PERMANOVA) (44). The enterotypes were generated by clustering samples with the Gaussian mixture model in the “Scikit-learn” Python package (version 0.22.1) (45), using the weighted UniFrac distance matrix.

### Inference of clade of Prevotella copri ASVs.

The reconstructed genome sequences of Prevotella copri by Tett et al. (12) were collected and converted into a BLAST database using the BLAST+ package (46). The sequence of each ASV was queried to the database using BLAST (version 2.9.0+) (46), and the reconstructed genomes containing the ASV sequence (perfect matches) were retrieved. The clades of these genomes were assigned as the clade of the ASV.

### Data sets from other countries.

To compare the Korean gut microbiome data with the data of healthy populations from other countries, we used 16S rRNA sequence data targeting the V3-V4 regions from the National Center for Biotechnology Information: BioProject PRJDB4360 (Japan) (15), PRJNA480547 (China, Beijing/Hangzhou) (13), PRJNA382861 (China, Shanghai) (14), PRJEB16755 (Chile) (17), and PRJNA350839 (Spain) (16). Data from Nigerian subjects were retrieved from MG-RAST (mgp83994) (18). We used only data from healthy adult samples, samples from healthy control groups (case-control studies), or “before” samples (intervention studies).

For the quality control, we checked the presence of two short sequences (5′-TATTGGACAATGGGCGC-3′ and 5′-CCTGTTCGCTCCCC-3′), instead of the primer sequences. These sequences are well conserved and located at the tips of the V3-V4 region, which enabled us to incorporate data sets for which the primer sequences were already trimmed off. Forward and reverse truncation lengths of 250 and 200, respectively, were applied during the sequence filtering step of the DADA2 pipeline.

### Population comparison analysis.

The data sets of the populations from South Korea and other countries were merged into a single data set, and ASV analysis, taxonomy annotation, and diversity analysis were performed together. The weighted UniFrac distance matrix generated from this single data set was used to compare the Korean gut microbiome with that of the populations from the other countries. The samples from other countries were designated *Bacteroides* ET or *Prevotella* ET based on the enterotypes of the Korean samples with the shortest UniFrac distance to them. The statistical difference of the *Bacteroides* ET ratios was evaluated using chi-squared tests, with the *chi2_contingency* function of the “SciPy” Python package (version 1.4.1) (47).

### Microbial cluster analysis.

The association between the pairs of ASVs was evaluated with respect to two aspects: whether they were statistically more or less frequently found together and whether they were quantitatively associated. For the evaluation of cooccurrence, Fisher’s exact test was performed using the contingency table with the numbers of samples in which the two ASVs were observed together, only one ASV was observed, and none was observed. Quantitative association was evaluated by calculating the *P* values for the Spearman correlation coefficients between the logarithm-transformed nonzero abundances of the two microbes. In the quantitative association analysis, only the samples with nonzero reads for both ASVs were used in order to highlight quantitative association. In cooccurrence and quantitative association analyses, only the ASVs that were observed in 10% or more of samples were used. The statistical significance of cooccurrence and quantitative association was defined at the false-discovery rate (FDR) of 10^−6^ and 10^−3^, respectively. A stringent statistical cutoff was applied for cooccurrence evaluation in order to keep a balance between the number of cooccurring and quantitatively correlated associations. The number of associations for the two statistical cutoffs is listed in Table S5. The “SciPy” Python package (version 1.4.1) was used for the statistical analysis (47).

10.1128/mSystems.00179-21.8 TABLE S5 The number of cooccurrent and quantitatively associated ASV pairs. Download Table S5, XLSX file, 0.01 MB. Copyright © 2021 Lim et al. 2021 Lim et al. https://creativecommons.org/licenses/by/4.0/ This content is distributed under the terms of the Creative Commons Attribution 4.0 International license.

We constructed a network using the cooccurring and quantitatively correlated ASV pairs as edges and the ASVs as nodes. We sought for the cliques with size 3, or three mutually connected nodes, and the connected cliques were merged into groups. We manually disjointed the groups to have the same phylum and joined the ASVs that are connected to an ASV with the same taxonomic label. The groups were then designated microbial clusters, and the clusters were labeled according to the taxonomic name of the most abundant ASVs.

### Prevalence difference among the demographic groups.

The statistical significance of a microbe being more or less prevalent in a specific demographic group was evaluated using the hypergeometric distribution. It was assumed that *n* samples in the demographic group were drawn from the entire data set with *N* samples, where *K* samples possess the microbe. The *P* value of finding *k* samples having the microbe was calculated. The statistical significance of the identified microbe-demographic group association was adjusted using the Benjamini-Hochberg method to control the FDR under 0.1. The “SciPy” Python package was used for the statistical analysis (47).

### Host factor association analysis.

**(i) Feature transformation.** Before the association analysis, outliers in the host factor metadata were identified and their values were replaced with the outlier cutoff values. For each numeric feature, the interquartile range (IQR) was calculated, and samples of which value is smaller than the first quartile minus 1.5 times IQR (lower cutoff) or larger than the third quartile plus 1.5 times IQR (upper cutoff) were regarded as outliers. The value of outliers was replaced with the lower and upper cutoff values accordingly. This transformation was employed to limit the influence of outlier data points on the final association outcome as the f-statistic based on the residual sum of squares was used to search for the associations.

**(ii) Association analysis.** The association between a host factor and an ASV abundance was evaluated by constructing a linear model predicting the value of the host factor with the abundance of the ASV and testing the statistical significance of the model with f-statistic (Wald test). The abundance of the ASV was log-transformed before analysis. When the abundance of the ASV was more strongly associated with age than a host factor, age and the host factor were both used as independent variables of the linear model. The statistical significance was then adjusted using the Benjamini-Hochberg method to control the FDR under 0.2. The “SciPy” Python package (version 1.4.1) was used in the statistical analysis (47), and the “Scikit-learn” Python package (version 0.22.1) was used to build linear models (45).

**(iii) Graphical representation of the feature association.** The ASV interaction network was converted into Cytoscape XML format files, with nodes representing microbes at the ASV level and with edges representing the quantitative associations, using in-house Python script (Python version 3.6.9). The statistical association of ASV abundance with each host factor was visualized as the color of the nodes. The microbial clusters and their association with host factors were illustrated with Cytoscape (version 3.8.0) (48).

### Ethics approval and consent to participate.

This study was approved by the Institutional Review Board (IRB) of Chung-Ang University Hospital (IRB number 1750-002-281). All participants gave written informed consent.

### Data availability.

The data set supporting the conclusions of this article is available in the European Nucleotide Archive repository, under accession no. PRJEB33905.
