# Supplementary figures and images for: Gut Microbiome Structure and Association with Host Factors in a Korean Population
Source: mSystems. 2021 Aug 3;6(4):e00179-21. doi: 10.1128/mSystems.00179-21 (PMC8407462; doi:10.1128/mSystems.00179-21)

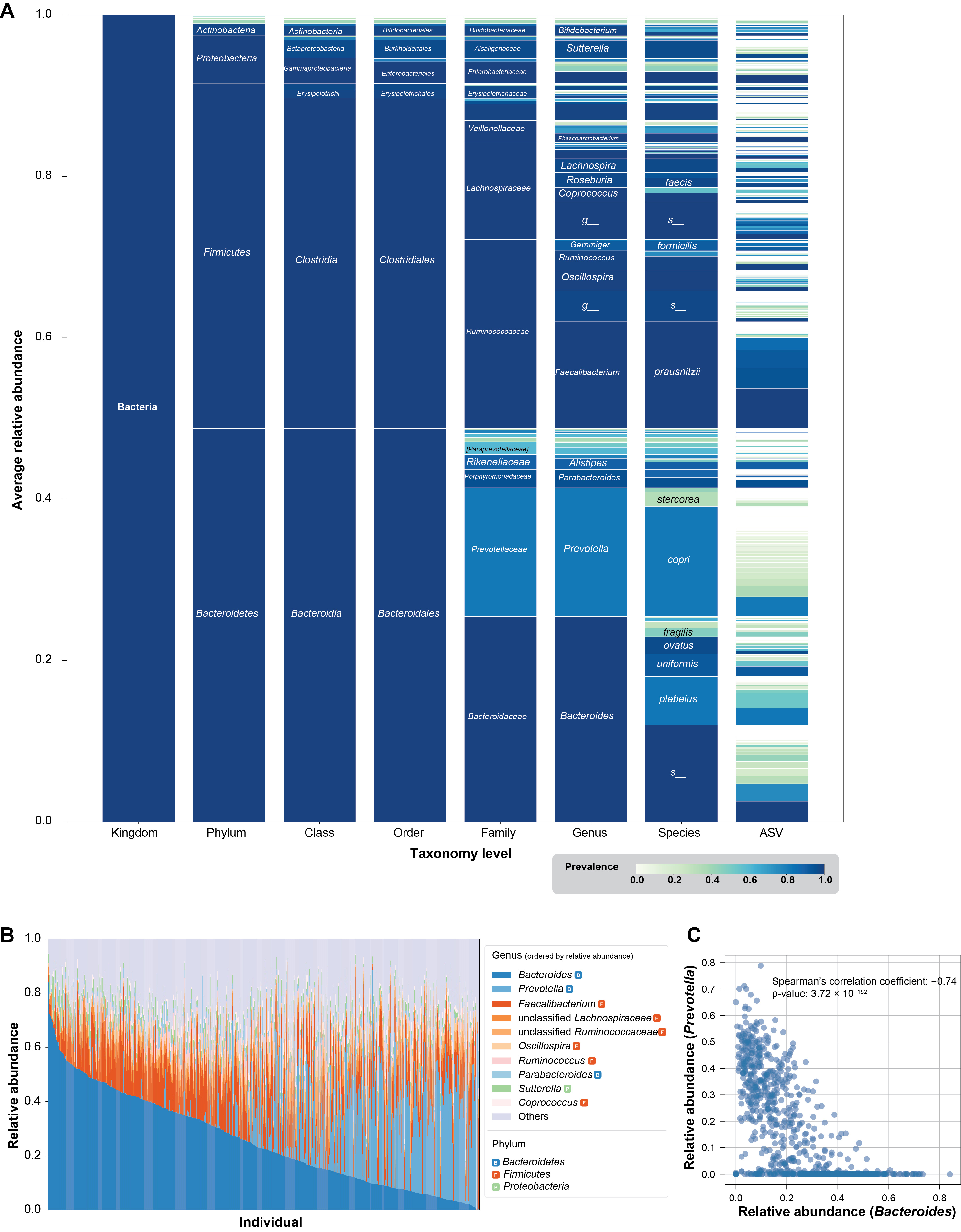

Supplement: FIG S1 [file msystems.00179-21-sf001.jpg]

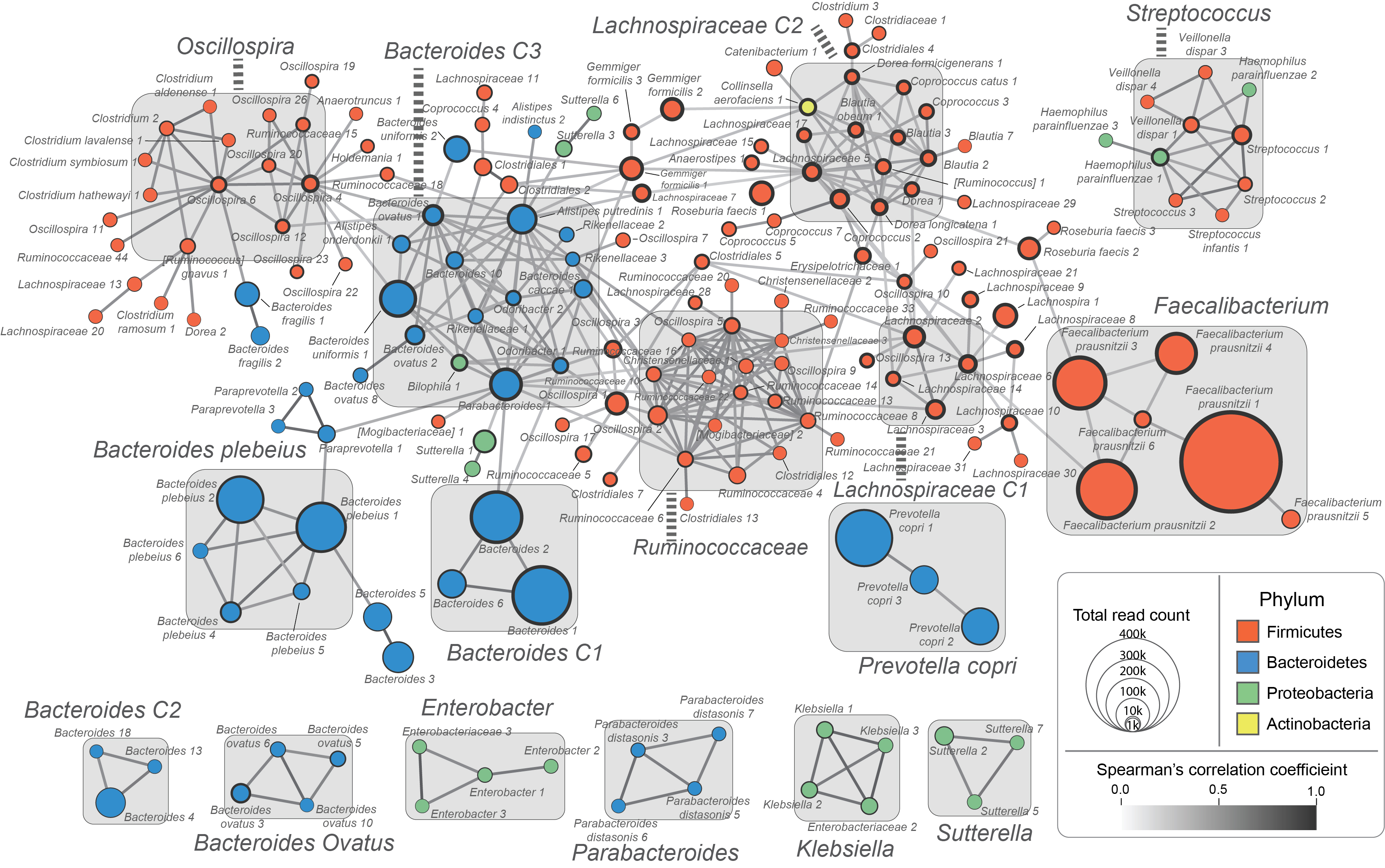

Supplement: FIG S2 [file msystems.00179-21-sf002.jpg]

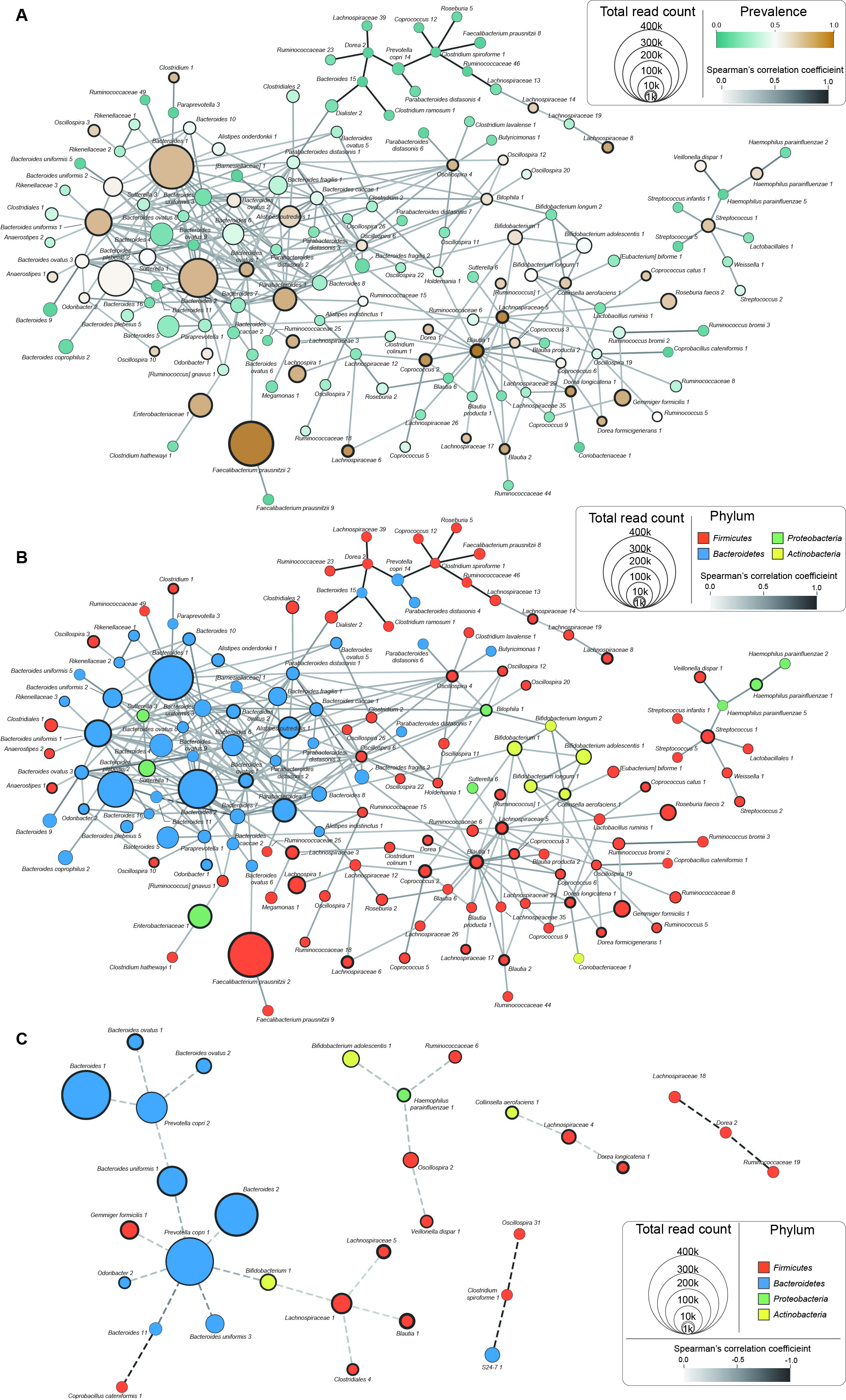

Supplement: FIG S3 [file msystems.00179-21-sf003.jpg]
